# Value Orientations and Institutional Trust as Contributors to the Adoption of Online Services in Youth: A Cross-Country Comparison

**DOI:** 10.3389/fpsyg.2022.887587

**Published:** 2022-05-16

**Authors:** Žan Lep, Aleš Trunk, Katarina Babnik

**Affiliations:** ^1^Department of Psychology, Faculty of Arts, University of Ljubljana, Ljubljana, Slovenia; ^2^Department of Economics, International School for Social and Business Studies, Celje, Slovenia

**Keywords:** individual values, cultural values, institutional trust, Internet use, e-services utilisation, youth, South-eastern Europe

## Abstract

Internet usage data from around the globe show that adolescents are the most frequent Internet users, but mostly for leisure activities and maintaining social contacts. In the present study, we focused on Internet use for e-services, which could improve youth efficiency in the financial domain (responsible consumer behaviour) and bridge the online divide in youth. Specifically, we explored how societal constructs (namely, institutional trust and personal values) influence the use of the Internet for online shopping, e-banking and communication with providers of goods and services online. We used a representative sample of adolescents (*N* = 10.902) from 10 countries of Southeast Europe where a great variability in Internet use is present, and where the use of e-services is generally lower than the EU average. This also allowed for meaningful cross-country comparisons. We tested a structural equation model of values predicting the use of the Internet through institutional trust (including some relevant demographic variables such as settlement size, SES and Internet use frequency) which was grounded in social capital theory, cultural theory and Schwartz human values model. The model exhibited a good fit to the data but the strengths of regressional paths were rather modest. Looking into the cross-country stability of the model, however, revealed some notable differences: while the relationship between trust and use of the Internet for e-services was modest in some countries, the relationship was insignificant in other countries, where Internet usage is lower in general. This suggests that strategies aimed at leveraging e-services and digital technology potential in youth should also account for cultural specificities in the transitional economies and cultural settings with sub-optimal adoption of digital services.

## Introduction

The Internet connects almost all parts of the world today, but not all regions and all groups of people are ‘connected’ equally. According to the International Telecommunication Union (ITU, 2020), about a half of the world’s population was using the Internet in the end of 2019, the year the data, used in this paper, pertains to. The population aged between 15 and 24 stands out in terms of Internet use, however, with 70% usage rate (ITU, 2020, p. 7). Internet use among young people is particularly high in developed parts of the world. In urban areas globally, 72% of households have access to the Internet, compared to only 37% in rural areas (ITU, 2020, p. 6). Internet access and usage are also growing rapidly in the European Union. In 2019, on average, 90% of households in the EU-27 had access to the Internet, with the highest percentage in Netherlands (98%) and the lowest in Bulgaria (75%). Nonetheless, Bulgaria, together with some other EU countries with comparably lower rates of Internet use, is characterised by a strong increase in household access to the Internet in the last observation period between 2014 and 2019 (Eurostat, 2020a). As on a global scale, daily Internet use in EU-27 is also most widespread in the age group between 16 and 29 years old with around 94% of adolescents and emerging adults using the Internet daily, compared to 77% of adults (Eurostat, 2020a).

The COVID-19 pandemic and the associated measures of social distancing, quarantine and closures of many activities have led to the increased use of the Internet, both for the maintenance of social contacts and usage of various electronic services. Specifically, its impact was pronounced in terms of further development and strengthening of e-commerce (ITU, 2020; OECD, 2020). The transition to the use of online services among consumers during the pandemic, however, does not necessarily imply that increased rate of online purchases and services among consumers will remain at the same level after the pandemic concludes. This has already been pointed out by both research (Al-Hattami, 2021) and professional articles (van Rijn, 2021). Moreover, predicting consumer behaviour requires an understanding of a variety of factors that may (help) influence that behaviour (Sunny and George, 2018) and go beyond current disruptions such as the pandemic. In an effort to improve the understanding of e-commerce use in youth—digital natives who will have an increasingly important role in future economy—the aim of this study was to investigate the role of socio-cultural factors in the frequency of use of online services such as e-banking, online shopping and active online communication with service providers (giving feedback, evaluating products or services) among young people aged 14–29 from 10 countries of Southeast Europe.

In contrast to the other age groups, young people are the most frequent users of the internet (ITU, 2020). Daily free time devoted to internet use in this age group has increased significantly in recent years (OECD, 2018). Most often, adolescents use the internet for leisure activities, such as downloading music or videos, watching videos, engaging in social networking and sending or receiving emails (Durkee et al., 2012; Eurostat, 2020b). For them, the internet is primarily a tool to communicate with each other and acquire various content (Durkee et al., 2012; Akar, 2015). The use of internet related to social media and entertainment decreases significantly with age and is at its highest in middle to late adolescence (15–19 years), in contrast to internet use related to work and information needs, which is lowest in this age group and highest between 20 and 54 years (Kalmus et al., 2011). Similarly, young people (aged between 16 and 29 years) use the internet to a lesser extent for engaging in various electronic services such as internet banking, job searching, sending job applications, selling goods and services, requesting a medical examination and participating in professional associations (Eurostat, 2020b), suggesting that daily internet use *per se* does not imply the use of various electronic services or functions. Among the listed e-service examples drawn from the Eurostat survey 2020b, young people use online banking most frequently (almost 60% of young people), while only about 20% of them use the internet to sell services and goods. Still, significant differences in the types of internet use for this purpose can be observed across the EU-27 countries (Eurostat, 2020a), suggesting that research into e-commerce activity in youth should not focus on single countries if it is to be widely applicable.

Nowadays, young people generally have access to technology and sufficient skills to use it, which enables them to connect with both friends and strangers with ease, and gives them access to a large amount of information and knowledge. The use of the internet, however, could have varied effects on individuals’ functioning. If it is used productive purposes, such as (exclusively) for study and learning, for example its use is positively associated with higher academic achievement (Kim et al., 2017). On the other hand, the use of internet also represents one of the main risk factors for worsened mental health (e.g., loss of self-control, bad temper and inability to focus), antisocial behaviours and abilities, relationship problems (Akar, 2015), the development of internet addiction and pathological internet use (Durkee et al., 2012). One of such forms is the compulsive use of the internet for specific purposes such as computer-mediated communication and social networking (van den Eijnden et al., 2008; Király et al., 2014). While youth use social networks to socialise, maintain and/or deepen relationships, and to self-express, which can have positive outcomes, social media is also used for evaluating others. Overexposure to negative evaluations online can lead to a range of psychological and relational problems, and social isolation (Primack et al., 2019), which might cancel out the positive aspects of (non-compulsive) use of the internet.

In terms of internet use for online services, the period between adolescence and emerging adulthood is crucial for the socialisation of responsible consumers (Shim et al., 2010). At this stage of life, young people acquire habits and behaviours that represent an efficient use of financial services and purchasing channels, which are likely to persist into the future (Shim et al., 2010) and have spill-over effects on their ‘offline’ activities. In the case of online banking, for example Xue et al. (2011) found that internet use increases customers’ banking activities, such as acquisition of banking products and bank transactions performance. Young people could therefore be one of the most important target groups for the implementation of the digital market strategy published by the European Commission (2015). While the global digital economy presents a positive challenge, it also has some pitfalls that require changes in social culture and everyday habits (Lyubimov and Cherny, 2021).

Because the use digital economy is linked with cultural specificities, the understanding of consumer behaviour in the digital market requires a multicultural approach. Cross-country comparison is not a new approach to studying the use of the Internet and online services. However, it is less common for such studies to use a multilevel approach that allows inferences to be made about the individual- and social-level factors and mechanisms that predict behaviour in the Internet environment. The goal of our study was to validate the predicting role of values and institutional trust in e-services use and to test the role of country-specific environments in this prediction model. The data analysis was conducted for 10 countries of South-eastern Europe: Albania, Bosnia and Herzegovina, Bulgaria, Croatia, Kosovo, Montenegro, North Macedonia, Romania, Serbia and Slovenia. While there are significant differences in internet use across these countries, in most, the active use of the internet for various e-services in the general population is lower compared to the EU-27 average (Eurostat, 2020a). For example in Germany, Netherlands, Sweden and Denmark, more than 80% of the population aged 16–74 ordered or purchased goods for personal use online in 2021, compared to 38% in Romania and 33% in Bulgaria. The situation is similar in Serbia, North Macedonia, Montenegro, and Bosnia and Herzegovina. Internet use among the general population for participation in social networks was average or even above the EU-27 average in the countries analysed in this study (Eurostat, 2020a).

### Predicting the Use of e-Services

Among the more common conceptual models for predicting online users’ behaviour are Davis’ Technology Acceptance Model (TAM; Davis and Venkatesh, 1996), which explains the use of information technologies as a direct consequence of the intention to use them. The intention is predicted by two key variables—perceived usefulness and perceived ease of use. Both variables, however, are influenced by external factors involving the user (e.g., computer self-efficacy) and the characteristics of ICT itself (e.g., system design; Davis and Venkatesh, 1996). The validity of TAM in predicting consumer use of modern technologies is confirmed by empirical findings, but the variables included in TAM are not sufficient to fully explain intention to use technology nor its actual use (Sunny and George, 2018). Especially in the international setting, TAM might be a less appropriate theoretical starting point given one of its main shortcomings is its lack of inclusion of variables describing the social context of technology use (Sunny and George, 2018). Therefore, many studies that predict consumers’ online behaviour, such as those that examine the use of online rating systems for online purchases (Klaus and Changchit, 2019), e-commerce (Guzzo et al., 2016), online shopping (Sharma and Bhatt, 2018), e-banking (Mousa et al., 2021) and the use of online pharmacies (Sampat and Sabat, 2020), extend TAM with other variables and models.

**Figure 1:**
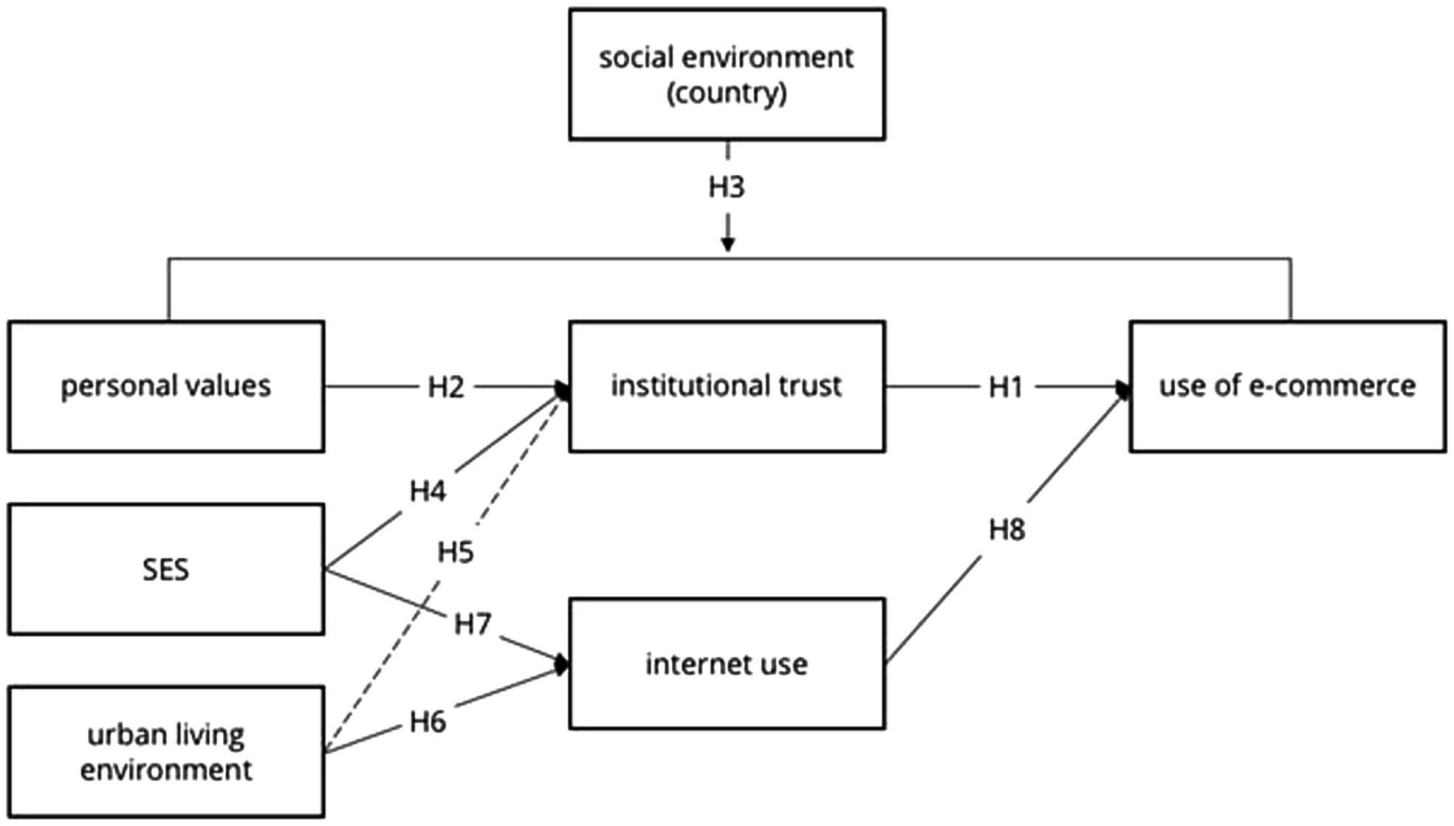
Theoretical model of the frequency of use of e-services among young people in different cultural environments.

In this study, we focused on two variables that predict consumer behaviour at the individual and social level, namely, trust and values. As noted by Rasty et al. (2021), trust is particularly important in online transactions because it reduces the uncertainty and risk associated with the ambiguity and inaccessibility of online offers and information. Trust, even in conjunction with perceived risk or uncertainty, has been shown to predict intention or actual use of different online services in models of online consumer behaviour prediction (Ashraf et al., 2014; Arshad et al., 2015; Guzzo et al., 2016; Muñoz-Leiva et al., 2017; Sunny and George, 2018; Sampat and Sabat, 2020; Xie et al., 2020; Al-Hattami, 2021). Values, on the other hand, have an important role in predicting the use of e-consumer services, especially in an international context (Zhang et al., 2018) because they represent abstract guidelines for decision-making and behaviour and are one of the fundamental elements of social culture. Regardless, to the best of our knowledge, neither trust, nor values have been explored as predictors of e-services use in youth. In the following paragraphs, we provide a summary of previous research on the role of trust and values in consumer behaviour, and present our proposed empirical model (Figure 1). The model is grounded in Schwartz’s value orientation theory (Schwartz, 2006, 2007), which describes and explains the development and impact of prevailing individual- and society-level value orientations on consumer behaviour.

### Trust: Specific, General, Social, and Institutional

Trust is the basis for building and maintaining relationships at the level of individuals, communities and societies (Kim and Kim, 2021). Like many other concepts addressed by different social sciences, the concept of trust has not yet been uniformly defined (Li, 2012, p. 101). Still, the multidisciplinary conceptualizations of trust (e.g., McKnight and Chervany, 2001; Li, 2007) provide a framework for its description and interpretation. As Li (2012) notes, most research on trust is based on defining it as an attitude, ‘a psychological willingness to accept vulnerability’ (Li, 2012, p. 101). Accordingly, in this study, we define trust as an attitude that includes not only the affective component (approval or disapproval) but also the cognitive component or belief (Devos et al., 2002). We further consider trust to be a relatively consistent tendency of individuals to accept risk and uncertainty in relation to different actors, as they attribute qualities such as ‘benevolence, integrity, competence, and predictability’ to them (McKnight and Chervany, 2001, p. 31). Similarly, Mishra and Mishra (2013) define trust as the willingness of one party to be vulnerable to another party based on the belief that the latter party possesses positive characteristics, such as reliability, competence and compassion, which might be especially applicable to online settings where the other party is less tangible.

Different scientific disciplines have various approaches to the interpretation of trust as they are interested in (among other things) trust as it pertains to different relationships and exchanges, such as trust in relation to known or unknown persons, individuals, groups or institutions. Furthermore, authors are considering different forms of trust, such as generalised trust (trust in unknown individuals or trust in a foreign group), institutional trust (trust in social institutions and the community; Kim and Kim, 2021) and specific trust related to a particular company, retailer, etc. (Kenning, 2008). Generalised (social) trust (Kim and Kim, 2021) is the belief that one can trust most people, even if they do not know them personally (Newton and Zmerli, 2011). In much of the previous work, generalised trust is interpreted as a personality trait that develops in early childhood and persists throughout life as a relatively stable trait (Kim and Kim, 2021). Despite the general assumption that generalised trust appears earlier in the development than other forms of trust and is a stable trait, determining the level of more specific and impersonal forms of trust, this is not confirmed in current studies. Sønderskov and Dinesen (2016), for example used two Danish individual-level panel datasets to show that institutional trust influences generalised trust. They conclude that generalised trust is a dynamic, changing phenomenon in which experiences with and trust in social institutions play an important role. The findings of this study are confirmed and complemented by Spadaro et al. (2020) who examined the hypothesis that institutional trust increases people’s trust in strangers (generalised trust) indirectly, *via* feelings of safety. Their research confirms that trust in social institutions strengthens trust in strangers through an increased sense of safety that enables people to accept vulnerability (Spadaro et al., 2020). Generalised trust thus (co-) arises when proper functioning of institutions is ensured and corruption and other forms of counterproductive societal behaviours are prevented (Sønderskov and Dinesen, 2016). When institutions fail to provide security to citizens, however, citizens look for other alternative ways to feel secure (Spadaro et al., 2020).

Studies on consumer behaviour have, for the most part, examined the role of specific trust (Kenning, 2008). For example, research on trust in technology or in the specific online service providers has found that this kind of trust mediates the relationship between various external and users’ internal factors in predicting the (intention to) use novel technological solutions for online consumption (e.g., Salo and Karjaluoto, 2007; Shiu et al., 2015; Guzzo et al., 2016; Muñoz-Leiva et al., 2017; Hallikainen and Laukkanen, 2018; Sunny and George, 2018). Particularly in the international context, however, specific trust may be a less appropriate variable to include in empirical models as this form of trust is highly context-dependent with a clear attitudinal object (Kenning, 2008) and specific determinants of attitudes towards it.

Another form of trust, playing an important role in the study of consumer behaviour, especially attitudes towards product safety (de Jonge et al., 2008), in supply chain perceptions (Sapp et al., 2009), intention or actual consumption (Pavlou and Gefen, 2004; Kalogeropoulos et al., 2019), is institutional trust. In the context of consumer behaviour, institutional trust can be defined as ‘trust built based on third-party structures’ (Pavlou and Gefen, 2004, p. 38). It is particularly relevant to online commerce, where consumers enter transactions with less familiar or unfamiliar suppliers, representing a broader institutional context of consumption (Pavlou and Gefen, 2004). Based on the evaluations of the ‘competence and fiduciary responsibility of institutional actors’ (Sapp et al., 2009, p. 530), consumers build their trust in social institutions, which in turn influences their consumer decisions and behaviour. As Devos et al. (2002, p. 484) further explain, ‘trusting an institution entail having confidence that the institution is reliable, observes rules and regulations, works well, and serves the general interest’. Institutional trust also plays an important role in trusting a particular sales product. Wu and Shen (2018), for example, find that institutional trust (trust in an online accommodation provider) determines trust in a particular accommodation product. Consistent with the empirically supported role of institutional trust in predicting consumer attitudes and behaviour (e.g., Pavlou and Gefen, 2004; de Jonge et al., 2008; Ricci et al., 2018; Carfora et al., 2019; ElKheshin and Saleeb, 2020) and the importance of institutional trust in citizens’ overall sense of security (Spadaro et al., 2020), we examined the role of institutional trust, expressed as trust in actors in the global economy (banks, IMF and corporations), in predicting the frequency of use of e-banking, online shopping and active online communication with providers of various services (giving feedback, evaluating products or services). Specifically, we expected that as:

*H1*: Young people’s institutional trust in global economy actors is positively related to the frequency with which they use electronic consumer services.

### The Role of Culture and Cultural Values in (Institutional) Trust

Several theories describe trust as an important element of social culture, among them the Social Capital Theory (Häuberer, 2011) and Cultural Theory (Douglas, 1992). The former defines social capital as ‘a property of relationships among individuals that are a resource actors can use and benefit from’ (Häuberer, 2011, p. 249). Thus, it is only present when there is trust in the exchange (Putnam, 2000; Häuberer, 2011). Cultural theory, on the other hand, provides a framework to explain the development of trust (as well as attitudes towards risk and uncertainty) as one of the fundamental components of social capital (Verweij, 2005). It assumes that the prevailing pattern of social relationships in society influences those relationships and their functioning (Conner et al., 2016). Social capital, therefore, is an individual and social asset that develops in informal and formalised (institutional) relationships (Häuberer, 2011). While the relationship between the two levels of social capital is complex, Kaasa (2019) states that the cultural context (cultural values) is more important than individual values in determining individual social capital.

Among the psychological theories that explain the role of individual and cultural values in the level of different forms of trust is (Schwartz’s, 2006, 2007, 2010, 2014a,b; Sagiv et al., 2011) theory of values. Schwartz (2006) interprets culture as a complex of meanings, beliefs, practises, norms and values. Prevailing values are a central and relatively stable feature of culture because they express ideas about what is good and desirable—cultural ideals that reflect and justify preferred social responses to the problems of collective life, such as boundaries between the individual and the collective, control of resource use and coordination of production among collective members (Schwartz, 2006). Individual values are relatively stable beliefs about desirable states or behaviours that guide individuals’ interpretations, decisions and behaviours across space and time (Sagiv et al., 2011).

Schwartz’s (1994) theory describes 10 individual values that are interconnected in the circumplex of bipolar dimensions: Openness to change (self-direction and stimulation) vs. Conservation (security, conformity and tradition); Self-transcendence (universalism and benevolence) vs. Self-enhancement (power and achievement); and Hedonism. The parallel of individual values is formed at the social level by three bipolar dimensions of culture that are potential solutions to the problems of preservation and development of the collective (Schwartz, 2006): Embeddedness (social order, obedience, respect for traditional and maintaining the status quo) versus Autonomy (intellectual and affective), Hierarchy (social power, authority, humility and wealth) versus Egalitarianism (equality, social justice, responsibility, help and honesty) and Mastery (ambition, success, daring and competence) versus Harmony (peace, unity with nature and protecting the environment). Societies emphasise one type of cultural pole and de-emphasise the other pole. For example, conservatism and hierarchical values are more important in Eastern than in Western Europe while egalitarianism, intellectual and affective autonomy, and mastery are less important in Eastern than in Western Europe (Schwartz et al., 2000).

Social culture determines and is reflected in the functioning of social institutions. Through their structure and functioning, social institutions convey the expected patterns of thoughts, feeling and behaviour to the members of the collective explicitly and implicitly, thereby mediating the impact of social culture (cultural values and normative system) on individuals (Schwartz, 2014a). Cultural values represent a broader social orientation based on which individuals form certain attitudes and ascribe intentions or motives for various social relationships (Kim and Kim, 2021). This influence, however, is neither direct nor generalised. Each person has their own role or an interweave of roles (Triandis and Suh, 2002) in the system that determines their unique position in society and thus the unique or individual-specific patterns of influence that social institutions exert on them. It is this uniqueness that determines the differences in values between individuals within society—‘They serve as filters that transform the same social experience into different subjective experiences for each individual’ (Schwartz, 2014a, p. 9). Therefore, phenomena that have both individual and social bases must be examined at both levels of analysis (Häuberer, 2011). Consistent with this assumption, Kim and Kim (2021), through a multilevel analysis of data from the World Values Survey (waves 5 and 6), found that individual values and (individual) demographic characteristics, as well as social variables (cultural values and characteristics of society), determine the level of generalised trust. They conclude that ‘social trust is not only an individual characteristic, but also a characteristic of society’ (Kim and Kim, 2021, p.14).

Based on the results of the European Social Survey, Schwartz (2007) reports that country-level differences account for 19% and individual-level differences account for 81% of the variability in the generalised trust index. At the individual level, trust in strangers is most strongly explained by education, security values (negative) and universalism (positive), and at the societal level by embeddedness (negative), egalitarianism and life expectancy (both positive). Hallikainen and Laukkanen (2018) found that culture explains 23% of the variance in consumers’ generalised trust and that this form of trust is an important predictor of perceptions of online trustworthiness. National culture, represented by cultural values and individual values, is thus an important predictor of generalised trust, and the role of cultural and individual values is also supported by research on institutional trust.

Devos et al. (2002) examined correlations between individual values and institutional trust, expressed as average ratings of trust in nine socially relevant institutions (e.g., education, politics, health, business and media). They found that values such as security, conformity, tradition and power were positively associated with institutional trust, while the associations with self-direction, universalism and hedonism were negative. A similar pattern of relationships between cultural values and institutional trust was found by Jamil and Baniamin (2020)—high authoritarian cultural orientation is positively associated with trust in public institutions. Marketing research also confirms the role of individual values (Shiu et al., 2015) and cultural values (Wu and Shen, 2018) in institutional trust in key actors of the economic system. For example, Cyr (2013) finds that (potential) users from eight countries evaluate various website design factors as a function of social culture (degree of uncertainty avoidance), degree of institutional trust and social capital, with social culture having stronger predictive power than economic and technological development factors in each country. Because individual values and cultural values, at the social level (Devos et al., 2002; Sagiv et al., 2011; Cyr, 2013; Shiu et al., 2015; Wu and Shen, 2018), predict the degree of institutional trust in different contexts (government, social and economic institutions and enterprises), we expected that as:

*H2*: Young people’s value orientations are related to their trust in actors in the global economy (banks, IMF and large corporations).

A review of prior research confirms the role of individual and cultural values in institutional trust. A review of research from different cultural settings shows that institutional trust plays an important role in consumer behaviour in different societies, both in consumer intention and actual consumption of services in a physical or online environment (Pavlou and Gefen, 2004; de Jonge et al., 2008; Moin et al., 2017; Wu and Shen, 2018; Carfora et al., 2019). Hypothesis 3 represents an extension of Hypothesis 2, as it implies the same relationships between the variables under study at the level of society as predicted by Hypotheses 1 and 2 at the level of the individual. This hypothesis is also partially grounded in the study of Morselli et al. (2012), in which the structure of the relationship between values and trust in institutions was found equivalent at the level of individuals and societies in student samples.

*H3*: Institutional trust in global economy actors mediates the relationship between young people’s individual values and their use of consumer e-services, regardless of their country of residence.

### Demographic Characteristics and the Use of e-Services

Numerous studies show that levels of trust in strangers (Kim and Kim, 2021) and trust in institutions (Schwartz, 2007) are influenced by various individual demographic variables such as age, gender, income or socio-economic status, education and social engagement. In this context, Schwartz (2007) finds that the strongest predictors of institutional trust are education, the fact that a person has never been unemployed, and income (all predictors were positive). We did not expect age to play a significant role in the sample of young people, nor the level of attained education, since the developmental period under study is still characterised by prolonged education and low variability in both variables. Like some previous research on the role of socio-economic status (SES) on institutional trust (Andreoni et al., 2021; Arvanitidis et al., 2021), we expected a positive relationship between SES and institutional trust among youth:

*H4*: Monthly family income is positively related to trust in global economic actors (banks, IMF and large corporations).

Research on attitudes towards the European Union and the EU Parliament examines the so-called urban–rural divide (Schoene, 2019; Scipioni and Tintori, 2021) and considers attitudes towards the EU Parliament and the EU as an institution to be a form of institutional trust. Even though institutional trust in EU institutions is stronger in larger cities, the place of residence is neither a strong predictor of negative attitudes towards the EU as an institution nor a strong predictor of the level of institutional trust (Schoene, 2019; Scipioni and Tintori, 2021). Because the degree of urbanisation does not directly determine the degree of institutional trust, likely because this relationship is more complex and co-determined by other factors (Scipioni and Tintori, 2021), we expect that as:

*H5*: Living in either a rural or urban environment is not significantly related to the level of institutional trust.

Nevertheless, the degree of urbanisation of the place of residence has a significant impact on internet use. Especially in rural areas, access to the internet may be disrupted or there may be no access to the internet at all. According to the ITU (2020), internet usage around the world is highly dependent on where people live—in particular, whether they live in an urban or rural environment. The lifestyle of young people living in rural areas also differs from those living in urban areas, but this relationship is not direct and is also explained by other social and cultural factors (Machado-Rodrigues et al., 2014). Similarly, SES may prevent or reduce the frequency of internet use as well as the use of e-consumer services among young people. Therefore, we included this aspect in the model shown in Figure 1:

*H6*: Young people living in rural areas are generally less likely to use the internet.

*H7*: Monthly family income is positively related to internet use.

Finally, hypothesis 8 (*H8*) assumes that “the overall frequency of internet use is significantly related to the frequency of use of e-consumer services by young people”. Although the frequency of internet use is determined by several factors already described (urban–rural living environment, SES, education level, etc.), the frequency of e-service use itself may be influenced by the fact that a person spends more or less time using the internet, regardless of demographic or structural factors.

## Methods and Measures

### Participants

Data for this paper were drawn from FES Youth Study Southeast Europe 2018/2019 dataset, which is publicly available for research purposes (Friedrich-Ebert-Stiftung, 2019). The full sample included 10.909 participants aged from 14 to 29 (*M* = 21.9, *SD* = 4.5). The sample sizes varied from 711 in Montenegro to 1,500 in Croatia. The full sample was balanced by gender (50.2% male) with 54.4% of participants enrolled in formal education (26.1% were secondary and 25.9% tertiary school students). Around a third of participants (30.9%) were employed full-time or self-employed (with further 8.8% employed part time or occasionally), and 26.8% reported living outside their parental home. The majority of participants (66.4%) noted they have the access to the internet ‘practically all the time’, with further 28.8% reporting access to the internet every day. On average, the participants reported spending 4.7 h per day online (*SD* = 3.6). Detailed demographic data on participants from each of the countries are presented in Supplementary material.

### Procedure

The dataset includes a sample of 10.909 participants from 10 countries of Southeast Europe: Albania, Bosnia and Herzegovina, Bulgaria, Croatia, Kosovo, Montenegro, North Macedonia, Romania, Serbia and Slovenia. The survey was implemented by local research agencies and institutes in the 10 countries based on representative randomised samples of youth between the ages of 14 and 29. The samples were stratified along key socio-demographic characteristics such as age, gender, place of residence and type of community.

The survey was developed in English and then translated into the local languages (Albanian, Bosnian, Bulgarian, Croatian, Serbian, Macedonian, Montenegrin, Romanian, Serbian and Slovenian) using a forward-backwards procedure. Data were collected during face-to-face interviews using the CAPI method (computer-assisted personal interviewing). The interviews consisted of an oral and a written part to fill out more intimate questions. The interviews lasted for around an hour on average, and the response rate varied from 38% in Kosovo to 83% in North Macedonia (see Lavrič et al., 2019 for more information on the study).

### Measures

#### Internet and e-Services Use

First, participants reported how regularly they have access to the internet (either Wi-Fi, mobile, public connection etc.). The frequency of internet use was then measured using an open-ended question; How *many hours a day on average do you spend on the Internet?* The data were recorded to eliminate inconsistencies in responses to both questions (e.g., responses of the 231 participants who have access to the internet less than once per week were fixed to zero) and outliers (responses larger than 24 h/day were recoded to missing variables).

The use of internet for e-services was assessed using three 3-point scales, where participants indicated how often they use the internet for online shopping, online banking and rating products or services, providing feedback or recommendations (1 – *never*, 2 – *sometimes* and 3 – *often*, *at least once per week*). Item non-responses and responses *I do not know* were coded as missing variables. While the three responses were directly modelled in structural equation model, the latent construct had good reliability (ω = 0.80, ranging from 0.72 to 0.86 across countries).

#### Personal Values

Within the FES Youth Study, values are measured both explicitly and implicitly using questions on basic value orientations, and, for example participants’ priorities when choosing a job. To operationalise the latent variables that roughly correspond to basic human values proposed by Schwartz Theory of Basic Values (Schwartz, 1992, 2012), we followed the procedure presented by Lep and Kirbiš (2021), who used a dataset stemming from the same study. In short, self-enhancement (power and achievement) was measured using the mean of five Likert-type indicators (*How important to you is: graduating from university*, *having a successful career, getting/being wealthy, wearing branded clothes* and *How important to you personally is having the feeling of achieving something when it comes to choosing a job?*). Self-direction was measured using two items (*How important to you is: taking responsibility, being independent*). Self-transcendence (universalism and benevolence) was measured by four items (*How important to you is: participating in civic actions, being faithful to partner, being faithful to friends* and *How important to you personally is the possibility to do something valuable for society when it comes to choosing a job*). Finally, conservation (tradition, conformity and safety) was measured using five items (*What young people need most of all is strict discipline by their parents, It is acceptable to cheat on taxes if you have a chance, It is acceptable to use connections to ‘get things done’, How important to you personally is job security when it comes to choosing a job* and *How important for a happy life is living in a good country*). The scales had sufficient internal consistency (self-enhancement: *H* = 0.79, self-direction: ρ = 0.56, self-transcendence: *H* = 0.65 and conservation: *H* = 0.85).

#### Institutional Trust

To measure specific institutional trust, we chose the items related to trust in institutions related to commercial activities, namely, big companies, banks and the International Monetary Fund (IMF). The participants indicated how far they trust each of them using a 5-point Likert-type scale (1 – *not at all*; 5 – *fully*). The variables were again included in the structural equation model independently, but the latent construct had good reliability (*H* = 0.81, ranging from 0.78 to 0.90 across countries).

#### Demographic Characteristics

Socio-economic status of each the participant’s family was assessed using a self-report item, *Which of the following descriptions most adequately describes financial situation in your household?* (1 – *We do not have enough money for basic bills (electricity, heating…) and food*; 5 – *We can afford to buy whatever we need for a good living standard*).

The settlement size in which the participants reside was operationalised as an ordinal variable, wherein the settlements were divided into five groups based on the number of inhabitants (under 2,000 inhabitants, 2,000–5,000, 10,000–20,000, 50,000–100,000 and more than 100,000 inhabitants).

### Analyses

All the hypotheses were tested within a structural equation model using R package lavaan (Rosseel, 2012). Because the distribution of scores on some of the scales deviated significantly from the normality, the robust maximum-likelihood estimator (MLR) was used. The cut-off values of RMSEA ≤0.06, SRMR ≤0.05, and CFI/TLI ≥ 0.95 (Marsh et al., 2004; Hooper et al., 2008) were considered indicative of a good model fit. After we established model fit using the full sample, we used multigroup SEM to test for its invariance across countries (constraining model parameters to be equal across countries). The reliabilities of the scales were assessed using Revelle’s ordinal omega for ordinal scales, Spearman-Brown coefficient for the self-direction scale with only two items (Eisinga et al., 2012), and *H*-coefficient (Hancock and Mueller, 2001), a measure of maximal reliability for an optimally weighted scale (McNeish, 2018), for the rest of the scales.

## Results

### The Relationships Between Observed Variables

To test our hypotheses, we first observed the pairwise correlations between the constructs of interest (see Table 1). The results for the SEE region at large showed that the size of the settlement participants resided in was modestly, but significantly related to institutional trust and internet use—the bigger was the settlement, the lower was institutional trust (H5) and the more frequently they used the internet, both in general and for e-services activities (H6). As anticipated (H7), higher socio-economic status of the respondents’ family was related to slightly higher amount of time dedicated to internet use, and more frequent use of internet in general was related to more frequent use of the internet for e-services (H8).

**Table 1:** Pairwise correlations between the observed constructs on the regional level.

Variable	(1)	(2)	(3)	(4)	(5)	(6)	(7)	(8)
(1) Settlement size	—							
(2) Family SES	0.04^***^	—						
(3) Internet use	0.07^***^	0.05^***^	—					
(4) Self-enhancement values	0.08^***^	0.08^***^	0.06^***^	—				
(5) Self-direction values	0.04^***^	0.07^***^	0.03^**^	0.28^***^	—			
(6) Self-transcendence values	0.03^***^	0.05^***^	0.01	0.39^***^	0.41^***^	—		
(7) Conservation values	0.09^***^	0.00	0.07^***^	0.07^***^	−0.01	−0.06^***^	—	
(8) E-services	0.06^***^	0.08^***^	0.10^***^	0.05^***^	0.00	0.04^***^	0.01	—
(9) Institutional trust	−0.07^***^	0.05^***^	−0.08^***^	0.13^***^	−0.01	0.10^***^	−0.01	0.01

** p < 0.*01*,

*** *p < 0*.*001*.

On the regional level, self-enhancement and self-transcendence values were positively correlated with institutional trust and more frequent engagement in e-services. It is of note, however, that the size of correlations was low, and not all were significant in each of the countries (correlation matrices for each country are presented in Supplementary material). Looking into country-level relationships between values and institutional trust, conservation values were related to higher institutional trust in Bosnia and Herzegovina, Montenegro and Slovenia, but negatively in Bulgaria, Croatia and Kosovo. Self-enhancement values were related to higher institutional trust in all of the countries, as were self-transcendence values with the exception of Slovenia, where the relationship was negative. Self-direction was related to higher institutional trust in Montenegro and to lower in Slovenia. The direction of other (significant) correlations was generally the same across the countries.

### The Structural Model of Pathways Towards e-Services Use

To explore the relationship between variables, we constructed a structural equation model in which socio-economic status of the family and living environment contribute to more frequent internet use, and personal values, socio-economic status of the family and living environment contribute to institutional trust in key financial players. In turn, we posited that institutional trust and internet use contribute to engagement in e-services (see Figure 1).

**Figure 2:**
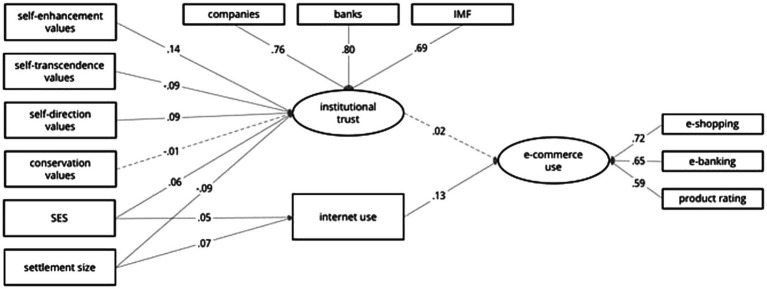
Structural model of the relationship between value orientations, institutional trust and frequency of internet use towards engagement in e-services on the regional level. All solid paths are significant at *p* < 0.001.

The model, presented graphically in Figure 2, fit the data well [*χ*^2^ = 683.53, *df* = 47, *p* < 0.001, CFI = 0.95, TLI = 0.94, RMSEA = 0.037, 90% *CI* (0.035, 0.040), SRMR = 0.028], and no corrections were applied. However, its predictive power was modest (*R*^2^ = 0.02 for e-services; see also Table 2). Higher SES contributed to more frequent internet use and higher institutional trust, while larger settlement size contributed to more frequent internet use, but lower institutional trust. Self-enhancement and self-direction values contributed to higher, while self-transcendence value orientation contributed to lower institutional trust. This in turn—contrary to our expectations—did not contribute statistically significantly to e-services use, which was positively predicted by more frequent internet use.

**Table 2:** Shares of explained variance (*R*^2^) for the endogenous variables in the model.

Country	Internet use	Institutional trust	e-Services
Overall	0.01	0.04	0.02
Albania	0.02	0.04	0.08
Bosnia and Herzegovina	0.01	0.04	0.01
Bulgaria	<0.01	0.04	0.10
Croatia	<0.01	0.07	0.03
Kosovo	0.02	0.07	0.04
North Macedonia	0.01	0.06	0.02
Montenegro	0.01	0.14	0.01
Romania	0.01	0.03	0.06
Serbia	0.01	0.05	<0.01
Slovenia	0.01	0.10	0.05

To explore the robustness of the model across countries, we continued by examining its fit using multigroup SEM. The figurative fit of the multigroup model was slightly worse but remained acceptable [*χ*^2^ = 1591.84, *df* = 470, *p* < 0.001, CFI = 0.92, TLI = 0.89, RMSEA = 0.050, 90% *CI* (0.047, 0.052), SRMR = 0.044]. However, constraining the parameters across countries to be equal resulted in poor fit to the data (Δ *χ*^2^ = 1292.9, *p* < 0.001). To test its partial invariance across countries, we proceeded by sequentially releasing and constraining each of the parameters. Still, no satisfactory fit was achieved, leading us to the conclusion that the strength of associations between variables varies across countries.

This approach revealed significant differences in variable associations across countries (the standardised regression coefficients for each of the countries are presented in Table 3). The relationship between the two demographic variables and internet use was only significant in Albania, Bosnia and Herzegovina, Kosovo, Montenegro and Romania. As predicted (H4), higher SES contributed to higher institutional trust in Albania, North Macedonia and Romania, while living in larger settlements generally contributed to lower institutional trust, which goes against our expectations (H5). Stronger self-enhancement values positively contributed to the institutional trust, but the strength of the relationship varied from medium in Slovenia to insignificant in some other countries. Self-transcendence values were negatively related to institutional trust in Slovenia and low-to-moderately positively in Albania, Croatia, North Macedonia, Montenegro and Serbia. In the structural model which—compared to bivariate correlations—accounts for some measurement error and controls for partial correlations between variables, self-direction values contributed to moderately lower institutional trust in Croatia, North Macedonia and Slovenia. These values, however, did not contribute to institutional trust in other countries. Finally, the relationship between conservation values and institutional trust varied from moderately positive in Montenegro to slightly negative in Kosovo. Even though both strength and direction of the relationships varied, value orientations of youth were related to their institutional trust towards key players in the global economy in all the countries but Romania, offering some support for our second hypothesis (H2). In line with our hypotheses, the institutional trust of the participants contributed to their e-services use (H1) in Croatia, North Macedonia and Slovenia, and mediated the contribution of personal value orientations in those countries (H3). Regardless, the shares of explained variance of e-services use remained low in all of the countries.

**Table 3:** Standardised regression coefficients in the multigroup structural model of the relationship between value orientations, institutional trust and frequency of internet use towards engagement in e-services across countries.

Path	Albania	BiH	Bulgaria	Croatia	Kosovo	North Macedonia	Montenegro	Romania	Serbia	Slovenia
Institutional trust → e-services	0.06	0.09	−0.06	0.12^***^	0.03	0.12^*^	0.05	−0.01	0.04	0.18^**^
Internet use → e-services	0.27^***^	0.04	0.32^***^	0.12^***^	0.19^***^	0.07	0.06	0.25^***^	0.04	0.14^**^
SE → institutional trust	0.02	0.05	0.10	0.09^*^	0.21^***^	0.07	0.13^*^	0.04	0.18^***^	0.22^***^
ST → institutional trust	0.15^***^	0.05	0.09	0.12^**^	0.04	0.13^**^	0.16^**^	0.07	0.09^*^	−0.14^**^
SD → institutional trust	−0.04	−0.07	−0.07	−0.26^***^	−0.05	−0.12^**^	0.04	−0.08	−0.05	−0.11
CO → institutional trust	−0.01	0.17^***^	−0.11^**^	−0.07^*^	−0.14^***^	0.05	0.24^***^	−0.04	0.02	0.09^*^
SES → institutional trust	0.12^**^	−0.04	0.05	0.03	0.04	0.09^*^	0.06	0.12^**^	0.05	0.04
Settlement size → instit. trust	−0.05	−0.07^*^	0.00	−0.15^***^	0.03	−0.14^***^	−0.03	−0.02	−0.08^*^	−0.13^***^
SES → internet use	0.13^***^	0.07^*^	0.02	0.01	0.11^**^	−0.07	0.11^*^	0.04	0.06	0.07
Settlement size → internet use	0.03	−0.03	−0.04	0.03	0.07^*^	0.03	−0.04	0.10^**^	0.04	0.01

SE, self-enhancement values; ST, self-transcendence values, SD, self-direction values and CO, conservation values.

* *p* < 0.05,

** *p* < 0.01,

*** *p* < 0.001.

## Discussion

The aim of this study was to explore how e-services use in youth across Southeast Europe is related to their values. Moreover, we were interested in whether this proposed association is mediated by the institutional trust in the actors of the global economy. Our choice to explore the proposed associations in a sample of adolescents and emerging adults was motivated by both the spikes in internet use in this age group (Kalmus et al., 2011; Durkee et al., 2012; Akar, 2015; Eurostat, 2020b), but also broader trends towards online economy, exacerbated by the ongoing COVID-19 pandemic (ITU, 2020; OECD, 2020), that could be considered as one of the positive uses of this technology (e.g., managing money more efficiently, improving access to goods and services). Youth, however, could be disadvantaged in using the internet in such a way, as they continue to learn about healthy financial functioning in those developmental periods (Shim et al., 2010, 2015) but are also burdened by their moral and identity development in the ideological domain (including values; Arnett, 2014; Walker and Iverson, 2016). Both aspects of their development before adulthood, namely, development of financial knowledge and skills, and of value orientations, were found to persist into adulthood to a certain degree (e.g., Shim et al., 2010; Vecchione et al., 2016) and influence their behaviour and various aspects of wellbeing (e.g., Sorgente and Lanz, 2019; Iannello et al., 2020).

While almost all the participants had access to the internet, earlier studies using the same dataset found differences in the purposes and motives of using the internet between young people with different backgrounds, which could lead to social inequalities (Tomanović, 2019). Due to the digital divide across, but also within the countries (ITU, 2020; Eurostat, 2020a,b), we stipulated that participants’ use of the internet in general, and specifically their use of the internet for e-services, will be influenced by their demographic characteristics and by the socio-economic standing of their families. We tested our hypotheses within a structural equation model that had a good fit to the data and supported the importance of expected pathways from values to institutional trust, from demographic characteristics to the frequency of internet use and from both internet use and institutional trust towards specific use of the internet for e-services. Upon observing the invariance of the model, we found that the regression paths deviated significantly across countries. This suggests that while the proposed pathways are robust, the specific relationships between values are more nuanced, and broad consideration of the results on a regional level would lead to misinterpretation and would fail to observe specific patterns in data. Consequently, we focus here on the differences rather than generalised observations of our model.

The results support our expectations regarding the role of demographic variables on the regional level, but not in each of the countries. It seems that the use of the internet is more dependent on the demographic characteristics (settlement size and SES) in countries with comparably lower BDP (i.e., Albania, Bosnia and Herzegovina, and Kosovo; United Nations, 2020), where the frequency of internet use tends to be lower compared to other sampled countries (see Supplementary material), and where a percentage of youth still report a lack of access to the internet (see also Tomanović, 2019). On the one hand, this points to the importance of considering both the digital divide and social capital of youth in such studies (in the region under study, for example the social capital of youth was predictive of the type of internet use, e.g., leisure vs. education; see Tomanović, 2019), but on the other, might lead to less stable conclusions in these countries. Regardless, the overall frequency of internet use was associated with more frequent e-service activities in Albania, Bulgaria, Croatia, Kosovo, Romania and Slovenia. It is of note, that apart from Albania and Kosovo, those countries are all part of the European Union and its open market, which might mean that youth have more options for online shopping and banking, as suggested by data on online banking penetration (Eurostat, 2022). This stipulation is also supported by the fact that e-services use tended to be higher in those countries (see Supplementary material).

While the association was not significant in all the countries, youth living in larger cities tended to trust the economic institutions less. The interpretation of this finding is likely multi-layered. The study looked at the countries that underwent major political and economic changes and growth in the last three decades (i.e., transition from some form of socialism or communism to market economy), and the changes were likely more pronounced in bigger cities and economic centres (Tsenkova, 2006). Moreover, adolescents and emerging adults form their ideological positions and critically evaluate various societal systems (Smith et al., 2011). In the bigger cities, where the activity of different institutions is more salient and visible, also through the work of governmental agencies and non-governmental organisations, the trust in institutions and positive evaluation of their actions could be less strong (e.g., Kääriäinen, 2007). On the other hand, higher SES was predictive of higher institutional trust only in Albania, North Macedonia and Romania. While this is expected based on some previous findings (Andreoni et al., 2021; Arvanitidis et al., 2021), the insignificant association in other countries could perhaps point to the developmental specificity of this association or its cultural dependency.

The model(s) further support our hypothesis that value orientations do not contribute directly to e-services use but are predictive of institutional trust (Devos et al., 2002; Sagiv et al., 2011; Cyr, 2013; Shiu et al., 2015; Wu and Shen, 2018) in key economic players. Specifically, higher self-enhancement values were predictive of higher trust in economic players in Croatia, Kosovo, Montenegro, Slovenia and Serbia. This is in line with previous findings (Devos et al., 2002; Schwartz, 2007) but also suggest that youth in the region might perceive these economic players as beneficial in fulfilling their values related to power and achievement, perhaps by enabling them to use their money more efficiently.

Likewise, self-transcendence values contributed to higher institutional trust in some countries (Albania, Croatia, North Macedonia, Montenegro and Serbia) but were predictive of lower trust in Slovenia. This points to the importance of considering the values as not entirely individual, but rather as a construct influenced by processes in the society at large (Schwartz, 2014a). While youth in most of the South-eastern European countries might perceive the economic players under study as positive influences, it seems that Slovenian youth do not trust them regarding the improvement of the lives of people in general or helping those in need.

Participants who value independence and taking responsibility for their own future (self-direction values) were, as found in previous studies (Devos et al., 2002; Schwartz, 2007), more wary and less trusting of the institutions under study, perhaps because they rely more on themselves to achieve their goals in life. Finally, the contribution of conservation values towards institutional trust varied. Bosnians, Montenegrins and Slovenians who valued tradition, conformity and safety reported higher, but Bulgarians, Croatians and Kosovars who valued tradition reported lower institutional trust. Again, this might suggest that adolescents and emerging adults from the former group of countries might perceive the institutions under study as drivers of stability, corroborating previous findings on the role of authoritarianism as cultural characteristic related to higher levels of institutional trust (Jamil and Baniamin, 2020). Conversely, in the latter group of countries, these institutions might be perceived as a threat to the established tradition and social order, for example by promoting ideas conflicting with those values. Moreover, these results might suggest that in some countries, the financial institutions under study might be perceived as contributing to better life, while in others, the role of the same institution might be perceived as opposite. Besides, the institutions under study might be perceived as foreign for respondents, and thus not influenced by their society and its values.

We also found some support for our expectations that institutional trust contributes to more frequent use of e-services and mediates the contribution of personal values. These paths were significant in Croatia, North Macedonia and Slovenia, but insignificant in other countries. This goes against our expectations regarding the stability of this relationship across countries. However, the significant relationship observed in Croatia, North Macedonia and Slovenia is in line with previous studies (Morselli et al., 2012), and the findings from the Macedonian sample, especially, mirror previous findings regarding the importance of trust as measured within the TAM model (Trenevska Blagoeva and Mijoska, 2017). It is also of note that Croatia and Slovenia are the only countries in the sample that are classified as high-income economies by the World Bank (United Nations, 2020; The World Bank, 2022), and both are members of the European Union, which might indicate that the e-commerce market is more developed and that youth might have better access to it. Indeed, Croatians and Slovenians reported the highest frequency of internet use.

### Limitations and Future Research Directions

Even though the model largely supported our hypotheses, its predictive power was modest even in countries where institutional trust contributed significantly towards the use of e-services, which might be explained by various factors. Perhaps most importantly, the frequency of e-services activity was low across all the participating countries, and the measurement scale was limited to three points. Both factors reduced the variability in the data, leading to potentially underestimated regression coefficients. This is likely also reflected in the data for Croatia and Slovenia, where the variability of e-services scores was highest, and the model held to the data. Thus, we stipulate that in samples where e-service activities are more prevalent, the relationships between the observed variables might be stronger. Because the data used in this study were collected before the pandemic, it would be informative to compare our findings with data, collected during or post-pandemic, as the pandemic has caused and increase in the use of internet for many daily activities, including shopping and baking (Al-Hattami, 2021).

As significant differences were observed in the model across countries, this enforces our initial stance that the behaviour of youth in the digital markets is influenced by their socio-cultural milieu. Moreover, institutional trust, and perhaps even more so their e-service activity, is likely highly contextualised in terms of market development, access to money and other financial instruments (e.g., credit cards), and the current economic and political environment in the country. Further studies would thus benefit from a more comprehensive model that would include not only demographic and psychological variables at the individual level, but also the characteristics of a country and its commercial, financial and banking sectors. In Slovenia, for example it is common for young people to have disposable income but also access to commission-free bank accounts with e-banking capabilities (see, for example Lep et al., 2021), enabling them to engage in e-banking and shop online. This might, however, not be the case in all the countries under study, considering vast differences in their economic standing (The World Bank, 2022). On a related note, it might be informative to widen the selection of institutional players in measuring institutional trust. This study focused on only three of the institutional players, which represent only a sample of all possible institutions that might influence participants’ internet use for commercial activities, or conversely that perhaps do not influence their trust at all—especially in the case of the International Monetary fund that might be less known and thus less important for youth in some countries under study (specifically those where this institution is less present in media and political discourse).

Besides the cross-cultural differences, both the stability and predictive power of the model could further be affected by the age of the participants, as younger participants are less likely to use the internet for e-services. Indeed, only around a tenth of the youth in the region report using the internet for e-services (see also Tomanović, 2019). Furthermore, their value orientations might be less stable (Vecchione et al., 2016), which, in turn, could lead to weaker relationships between the still-developing and changing constructs, which in turn lowers the predictive power of the model—an issue observed in our study. As the study focused on cultural influences on the e-services activity, exploring age differences within adolescence and emerging adulthood was outside the scope of the paper, but could and should be addressed in future studies on the topic. Furthermore, considering age alone would likely not be informative as such approach would not account for the differences between age cohorts (e.g., youth today were socialised in the internet-adjacent society and might thus accept online financial pathways more readily; Alhabash et al., 2015) and the process of adulting, which differs between countries (Arnett and Galambos, 2003). Because the perceptions of adulthood are culture-dependent, merely observing the differences between age groups might lead to more superficial conclusions.

## Conclusion

Public health measures such as quarantines and social restrictions during the COVID-19 pandemic led to the increased use of e-services among the population, further strengthening the idea of a global digital market. The analysis of data from the FES Youth Study Southeast Europe 2018/2019 supported the findings of some previous studies, indicating that variables determined by the social context (social culture) play an important role in explaining the use of e-services in the international sphere. The frequency of internet use plays a direct role in determining e-services use, while value orientations of young people, the SES of their family and the size of their settlement were indirectly related to the frequency of their e-services use, with trust in global economy actors (banks, IMF and large corporations) mediating this relationship. The results have revealed, however, that the model of expected relationships between variables is not stable in all studied countries, but only in some. This result is particularly important for practical purposes. In creating an effective digital single market strategy, which is also one of the objectives of the European Commission (2015), both a top-down approach (elements of development common to all countries) and a bottom-up approach (specific role of certain elements in each country) should be followed simultaneously. The results of the analysis we conducted with a sample of young people confirm that a completely uniform strategy cannot be effective; the specificities of each country should thus be identified and considered.

## Data Availability Statement

Publicly available datasets were analyzed in this study. This data can be found at: https://www.fes-soe.org/features/youth-studies/.

## Ethics Statement

Ethical review and approval was not required for the study on human participants in accordance with the local legislation and institutional requirements. Written informed consent to participate in this study was provided by the participants’ legal guardian/next of kin.

## Author Contributions

KB and ŽL conceived of the study. ŽL, AT, and KB conducted the literature review. ŽL conducted the analyses. All authors contributed to the interpretation of the data, drafted the manuscript, reviewed it for important intellectual content, and approved it for publication.

## Funding

The publication of the paper was financially supported by the Slovenian Research Agency within the research programme *Applied Developmental Psychology* (research core funding no. P5-0062).

## Conflict of Interest

The authors declare that the research was conducted in the absence of any commercial or financial relationships that could be construed as a potential conflict of interest.

## Publisher’s Note

All claims expressed in this article are solely those of the authors and do not necessarily represent those of their affiliated organizations, or those of the publisher, the editors and the reviewers. Any product that may be evaluated in this article, or claim that may be made by its manufacturer, is not guaranteed or endorsed by the publisher.
