# Supplementary material for: Value Orientations and Institutional Trust as Contributors to the Adoption of Online Services in Youth: A Cross-Country Comparison
Source: Front Psychol. 2022 May 16;13:887587. doi: 10.3389/fpsyg.2022.887587 (PMC9149369; doi:10.3389/fpsyg.2022.887587)
Supplement: Supplementary file 1 [file Data_Sheet_1.pdf]

Supplementary Material

to

Value orientations and institutional trust as contributors to the adoption of online services in youth: A cross-country comparison

frontiers

1 Demographic characteristics

Table 1. *Selected demographic characteristics by the country subsamples*

	Albania (*n* = 1210)		BiH (*n* = 1000)		Bulgaria (*n* = 1016)		Croatia (*n* = 1500)		Kosovo (*n* = 1200)		N. Macedonia (*n* = 1038)		Montenegro (*n* = 711)		Romania (*n* = 1048)		Serbia (*n* = 1170)		Slovenia (*n* = 1015)	
	n	%	*n*	%	*n*	%	*n*	%	*n*	%	*n*	%	*n*	%	*n*	%	*n*	%	*n*	%
Gender																				
Male	594	49.1	513	51.3	497	48.9	766	51.1	560	46.7	521	50.2	366	51.5	514	49.0	598	51.1	497	49.0
Female	616	50.9	487	48.7	519	51.1	734	48.9	640	53.3	517	49.8	345	48.5	535	51.0	570	48.7	518	51.0
Access to the internet																				
Not at all	35	2.9	12	1.2	15	1.5	5	0.3	19	1.5	3	0.3	1	0.1	40	3.8	5	0.4	10	1.0
Less than once/week	14	1.1	8	0.8	1	0.1	5	0.4	19	1.6	11	1.1	6	0.8	9	0.8	7	0.6	6	0.6
At least once/week	35	2.9	25	2.5	15	1.5	22	1.5	17	1.4	37	3.6	10	1.4	14	1.3	14	1.2	18	1.8
(Almost) every day	424	35.0	252	25.2	185	18.2	564	37.6	456	38.0	186	17.9	228	32.1	308	29.4	354	30.3	184	18.1
All the time	703	58.1	703	70.3	796	78.3	900	60.0	688	57.4	791	76.2	461	64.8	677	64.5	736	62.9	786	77.4
Living arrangement																				
With parents	939	77.6	774	77.4	664	65.3	1065	71.0	967	80.6	795	76.6	490	68.9	699	66.7	800	68.4	661	65.1
Out of parental home	261	21.5	226	22.6	346	34.1	432	28.8	232	19.3	233	22.4	192	27.0	338	32.4	333	28.6	345	34.0
Educational status																				
High school student	402	33.2	267	26.7	263	25.8	324	21.6	299	25.0	232	22.4	174	24.5	220	21.0	320	27.4	343	33.8
Undergraduate student	153	12.7	180	18.0	144	14.2	312	20.8	342	28.5	239	23.0	187	26.3	145	13.8	299	25.6	237	23.4
Master, postgraduate	48	4.0	22	2.2	49	4.8	75	5.0	49	4.1	59	5.7	53	7.5	29	2.7	106	9.1	105	10.3
Other	23	1.9	27	2.7	0	0.0	37	2.5	9	0.8	54	5.2	17	2.4	18	1.7	23	2.0	48	4.7
Not in education	543	44.9	469	46.9	544	53.5	728	48.5	447	37.3	362	34.9	218	30.7	594	56.7	312	26.7	215	21.2
Employment status																				
Full-time	268	22.1	250	25.0	472	46.5	592	39.6	221	18.4	296	28.5	226	31.8	462	44.1	336	28.6	243	24.0
Part-time	31	2.5	23	2.3	33	3.2	37	2.4	40	3.3	83	8.0	28	3.9	22	2.2	46	3.9	39	3.9
Occasional work	44	3.6	62	6.2	31	3.1	60	4.0	26	2.2	72	6.9	52	7.3	37	3.5	80	6.8	111	10.9
Not employed	788	65.1	628	62.8	118	11.5	736	49.0	888	73.9	466	44.9	318	44.7	407	38.9	513	43.8	472	46.5
Other	52	4.3	2	0.2	363	35.7	8	0.6	0	0.0	14	1.3	35	4.9	67	6.4	66	5.6	66	6.5

Notes. Sample sizes can differ between variablles due to missing values.

2 Means and correlations between variables under study

Table 2. *Means, standard deviations, and correlations between the studied variables in Albania*

	M	*SD*	(1)	(2)	(3)	(4)	(5)	(6)	(7)	(8)
(1) Settlement size	4.23	1.66	—							
(2) SES	3.24	0.96	.20***	—						
(3) Internet use	3.86	2.17	.05	.14***	—					
(4) Self-enhancement	4.31	0.66	-.03	.11***	.10***	—				
(5) Self-direction	4.55	0.71	.11***	.00	.01	.17***	—			
(6) Self-transcendence	4.28	0.59	.01	.03	-.07*	.33***	.30***	—		
(7) Conservation	3.89	1.00	.05	.06*	.13***	.07*	-.01	-.06*	—	
(8) E-services use	1.36	0.47	.06*	.15***	.23***	.15***	.11***	.11***	.03	—
(9) Institutional trust	2.68	1.10	-.03	.09**	-.03	.06*	.02	.13***	-.01	.03***

Notes. * $p < .05$, ** $p < .01$, *** $p < .001$.

Table 3. *Means, standard deviations, and correlations between the studied variables in Bosnia and Herzegovina*

	M	*SD*	(1)	(2)	(3)	(4)	(5)	(6)	(7)	(8)
(1) Settlement size	2.83	2.09	—							
(2) SES	3.63	0.79	.03	—						
(3) Internet use	4.81	3.23	-.02	.06	—					
(4) Self-enhancement	3.94	0.64	-.01	.10**	.00	—				
(5) Self-direction	4.65	0.51	-.05	.11***	.03	.22***	—			
(6) Self-transcendence	4.18	0.53	.02	.14***	.02	.43***	.40***	—		
(7) Conservation	3.71	0.87	.02	-.02	.05	-.09**	.10***	-.01	—	
(8) E-services use	1.48	0.54	.11***	.06	.04	.07*	-.12***	.05	-.16***	—
(9) Institutional trust	2.73	0.97	-.07*	-.05	-.10**	.01	-.02	.02	.16***	.09**

Notes. * $p < .05$, ** $p < .01$, *** $p < .001$.

Table 4. *Means, standard deviations, and correlations between the studied variables in Bulgaria*

	M	*SD*	(1)	(2)	(3)	(4)	(5)	(6)	(7)	(8)
(1) Settlement size	4.97	2.59	—							
(2) SES	3.32	0.97	.08*	—						
(3) Internet use	4.22	3.41	-.04	.01	—					
(4) Self-enhancement	4.12	0.65	.26***	.20***	-.04	—				
(5) Self-direction	4.65	0.57	.16***	.18***	-.07*	.32***	—			
(6) Self-transcendence	4.12	0.64	.04	.16***	-.09**	.46***	.41***	—		
(7) Conservation	4.28	1.01	.00	-.09**	.09**	-.01	-.12***	-.16***	—	
(8) E-services use	1.58	0.50	.12***	.05	.28***	.16***	.10**	.13***	.03	—
(9) Institutional trust	2.94	1.07	.01	.06	-.24***	.14***	.03	.14***	-.11***	-.12***

Notes. * $p < .05$, ** $p < .01$, *** $p < .001$.

Table 5. *Means, standard deviations, and correlations between the studied variables in Croatia*

	M	*SD*	(1)	(2)	(3)	(4)	(5)	(6)	(7)	(8)
(1) Settlement size	3.49	2.45	—							
(2) SES	3.74	0.79	.02	—						
(3) Internet use	3.47	2.85	.03	.00	—					
(4) Self-enhancement	3.73	0.67	.03	.22***	.14***	—				
(5) Self-direction	4.33	0.73	-.01	.16***	.06*	.37***	—			
(6) Self-transcendence	3.80	0.62	.02	.16***	.04	.42***	.50***	—		
(7) Conservation	4.19	0.92	.05*	-.10***	.06*	.09***	-.01	-.04	—	
(8) E-services use	1.74	0.57	.07**	.02	.08**	.02	-.08**	.07*	.04	—
(9) Institutional trust	2.47	0.91	-.11***	.02	-.03	.04	-.13***	.02	-.05*	.07*

Notes. * $p < .05$, ** $p < .01$, *** $p < .001$.

Table 6. *Means, standard deviations, and correlations between the studied variables in Kosovo*

	M	*SD*	(1)	(2)	(3)	(4)	(5)	(6)	(7)	(8)
(1) Settlement size	3.58	2.08	—							
(2) SES	3.74	0.87	.08**	—						
(3) Internet use	4.08	2.48	.08*	.12***	—					
(4) Self-enhancement	4.21	0.61	.06*	.19***	.09**	—				
(5) Self-direction	4.57	0.66	.05	.09**	.09**	.34***	—			
(6) Self-transcendence	4.16	0.53	.07*	.04	.00	.36***	.33***	—		
(7) Conservation	3.59	0.86	.08**	.00	.09**	.05	-.01	.02	—	
(8) E-services use	1.35	0.41	.07*	.08**	.15***	.12***	.08**	.09**	-.03	—
(9) Institutional trust	2.90	1.11	.03	.05	-.11***	.19***	.05	.09**	-.11***	.00

Notes. * $p < .05$, ** $p < .01$, *** $p < .001$.

Table 7. *Means, standard deviations, and correlations between the studied variables in North Macedonia*

	M	*SD*	(1)	(2)	(3)	(4)	(5)	(6)	(7)	(8)
(1) Settlement size	4.70	2.10	—							
(2) SES	3.65	0.93	.05	—						
(3) Internet use	6.36	4.05	.03	-.07*	—					
(4) Self-enhancement	4.09	0.71	.06	.10**	-.03	—				
(5) Self-direction	4.56	0.74	.02	.06*	.00	.38***	—			
(6) Self-transcendence	4.14	0.66	-.01	.05	-.01	.34***	.41***	—		
(7) Conservation	3.82	0.93	.09**	.13***	.05	.10***	.01	-.03	—	
(8) E-services use	1.69	0.59	.11***	.18***	.04	.16***	-.03	.07*	.01	—
(9) Institutional trust	2.54	1.10	-.11***	.09**	-.05	.09**	-.02	.10**	.06	.08*

Notes. * $p < .05$, ** $p < .01$, *** $p < .001$.

Table 8. *Means, standard deviations, and correlations between the studied variables in Montenegro*

	M	SD	(1)	(2)	(3)	(4)	(5)	(6)	(7)	(8)
(1) Settlement size	4.89	1.71	—							
(2) SES	3.60	1.05	$.16^{***}$	—						
(3) Internet use	6.62	4.88	.00	$.12^{**}$	—					
(4) Self-enhancement	3.98	0.74	-.03	.05	$.10^{*}$	—				
(5) Self-direction	4.62	0.64	.01	.00	-.05	$.21^{***}$	—			
(6) Self-transcendence	4.11	0.68	$.08^{*}$	-.02	.01	$.36^{***}$	$.40^{***}$	—		
(7) Conservation	4.39	1.25	$-.14^{***}$	-.06	.07	$.21^{***}$	.04	.00	—	
(8) E-services use	1.44	0.50	-.01	.02	.06	$.09^{*}$	.04	.00	$-.09^{*}$	—
(9) Institutional trust	2.46	1.25	-.02	.03	-.01	$.22^{***}$	$.14^{**}$	$.22^{***}$	$.22^{***}$	.04

Notes. * $p < .05$, ** $p < .01$, *** $p < .001$.

Table 9. *Means, standard deviations, and correlations between the studied variables in Romania*

	M	SD	(1)	(2)	(3)	(4)	(5)	(6)	(7)	(8)
(1) Settlement size	4.50	2.30	—							
(2) SES	3.42	1.11	$.18^{***}$	—						
(3) Internet use	4.56	3.25	$.12^{***}$	.05	—					
(4) Self-enhancement	4.03	0.70	$.23^{***}$	$.16^{***}$	$.16^{***}$	—				
(5) Self-direction	4.62	0.62	.02	$.13^{***}$	.06	$.30^{***}$	—			
(6) Self-transcendence	4.02	0.65	$.08^{*}$	$.10^{**}$	.01	$.33^{***}$	$.38^{***}$	—		
(7) Conservation	4.28	0.95	.05	.02	$.07^{*}$	$.14^{***}$	.04	-.01	—	
(8) E-services use	1.57	0.52	$.21^{***}$	$.15^{***}$	$.20^{***}$	$.18^{***}$	.03	$.13^{***}$	-.03	—
(9) Institutional trust	2.29	1.03	.00	$.10^{**}$	-.03	.06	.00	$.08^{*}$	-.01	-.04

Notes. * $p < .05$, ** $p < .01$, *** $p < .001$.

Table 10. *Means, standard deviations, and correlations between the studied variables in Serbia*

	M	*SD*	(1)	(2)	(3)	(4)	(5)	(6)	(7)	(8)
(1) Settlement size	5.43	1.91	—							
(2) SES	3.52	0.95	.07*	—						
(3) Internet use	5.66	4.57	.02	.06	—					
(4) Self-enhancement	3.77	0.68	.05	-.01	.11**	—				
(5) Self-direction	4.59	0.57	-.02	.01	-.06*	.19***	—			
(6) Self-transcendence	4.08	0.58	-.04	-.01	.01	.32***	.41***	—		
(7) Conservation	4.02	1.03	.03	.07*	.06	.11***	-.03	-.05	—	
(8) E-services use	1.49	0.51	.06*	-.03	.04	.13***	.05	.14***	-.02	—
(9) Institutional trust	2.23	1.02	-.08*	.05	-.04	.15***	.02	.12***	.03	.03

Notes. * $p < .05$, ** $p < .01$, *** $p < .001$.

Table 11. *Means, standard deviations, and correlations between the studied variables in Slovenia*

	M	*SD*	(1)	(2)	(3)	(4)	(5)	(6)	(7)	(8)
(1) Settlement size	2.76	2.31	—							
(2) SES	3.76	0.89	.00	—						
(3) Internet use	4.56	3.32	.00	.06	—					
(4) Self-enhancement	3.66	0.63	-.07*	.08*	.07*	—				
(5) Self-direction	4.63	0.58	.08*	.07*	-.04	.22***	—			
(6) Self-transcendence	4.08	0.56	.00	.02	-.01	.35***	.41***	—		
(7) Conservation	3.64	0.99	.05	.15***	.00	.19***	.00	-.05	—	
(8) E-services use	1.72	0.52	.02	-.05	.11***	.10**	.07*	.10**	.12***	—
(9) Institutional trust	2.53	0.90	-.12***	.06	-.04	.15***	-.13***	-.09**	.12***	.11***

Notes. * $p < .05$, ** $p < .01$, *** $p < .001$.
